# TORC2 is required for the accumulation of γH2A in response to DNA damage

**DOI:** 10.1016/j.jbc.2024.107531

**Published:** 2024-07-04

**Authors:** Adiel Cohen, Lea Lubenski, Ava Mouzon, Martin Kupiec, Ronit Weisman

**Affiliations:** 1 Department of Natural Sciences, The Open University of Israel, Ranana, Israel; 2 The Shmunis School of Biomedicine & Cancer Research, Tel Aviv University, Tel Aviv, Israel

**Keywords:** TOR complex (TORC), *Schizosaccharomyces pombe*, DNA damage response, histone modification, histone H2A phosphorylation

## Abstract

TOR protein kinases serve as the catalytic subunit of the TORC1 and TORC2 complexes, which regulate cellular growth, proliferation, and survival. In the fission yeast, *Schizosaccharomyces pombe*, cells lacking TORC2 or its downstream kinase Gad8 (AKT or SGK1 in human cells) exhibit sensitivity to a wide range of stress conditions, including DNA damage stress. One of the first responses to DNA damage is the phosphorylation of C-terminal serine residues within histone H2AX in human cells (γH2AX), or histone H2A in yeast cells (γH2A). The kinases responsible for γH2A in *S. pombe* are the two DNA damage checkpoint kinases Rad3 and Tel1 (ATR and ATM, respectively, in human cells). Here we report that TORC2-Gad8 signaling is required for accumulation of γH2A in response to DNA damage and during quiescence. Using the TOR-specific inhibitor, Torin1, we demonstrate that the effect of TORC2 on γH2A in response to DNA damage is immediate, rather than adaptive. The lack of γH2A is restored by deletion mutations of transcription and chromatin modification factors, including loss of components of Paf1C, SAGA, Mediator, and the bromo-domain proteins Bdf1/Bdf2. Thus, we suggest that TORC2-Gad8 may affect the accumulation of γH2A by regulating chromatin structure and function.

The Target of Rapamycin (TOR) protein belongs to the protein family of phosphatidylinositol-3-kinase-related kinases (PIKKs). TOR proteins are best known for their roles in regulating cellular growth, proliferation, differentiation, and survival (1, 2). The PIKK family includes five members with serine/threonine protein kinase activity: ATM (*a*taxia *t*elangiectasia *m*utated), ATR (*AT*M and *R*AD3-related), DNA-PK (DNA-dependent protein kinase), TOR, and SMG-1. ATM, ATR, and DNA-PK are critical for DNA damage responses, while SMG-1 is important for mRNA quality control (3, 4).

TOR kinases serve as the catalytic subunit of two structurally and functionally distinct and conserved complexes, termed TOR complex 1 (TORC1) and TOR complex 2 (TORC2). The fission yeast, *Schizosaccharomyces pombe*, contains two TOR homologs, Tor1 and Tor2, which were named based on the order of their discovery (5). Tor2 interacts with the Raptor-like subunit Mip1 to form TORC1, while Tor1 interacts with the Rictor-like subunit Ste20, and with Sin1 (mSin1 in human cells) to form TORC2 (6, 7).

Under optimal growth conditions, *S. pombe* cells proliferate predominantly as haploid cells. Starvation signals, in particular nitrogen-starvation, induce cells to enter quiescence (also known as the stationary phase), or to enter the sexual development pathway in the presence of opposite mating-type cells (8, 9). *S. pombe* TOR complexes are involved in the regulation of both growth and starvation responses. *S. pombe* TORC1 (hereafter simply referred to as TORC1) is an essential complex that positively regulates growth, while inhibiting starvation responses, including autophagy and sexual development. Accordingly, cells disrupted for TORC1 function arrest growth as small cells with a phenotype of nitrogen-starved cells and can readily enter the sexual development pathway (7, 10, 11, 12, 13). *S. pombe* TORC2 (hereafter referred to simply as TORC2) is not an essential complex, but cells lacking TORC2-Gad8 activity display a delay in entrance into mitosis (14, 15). Notably, TORC2-Gad8 becomes essential under many different stresses, including osmotic, oxidative, temperature, DNA damage, or DNA replication stress (5, 14, 16, 17, 18). In striking contrast to TORC1, TORC2 positively regulates autophagy and is absolutely required for sexual development (5, 13). The opposite roles of TORC1 and TORC2 in response to nitrogen starvation are coordinated by the action of the type-2A phosphatase complex PP2A-Pab1 (19, 20, 21). TORC2 regulates most of its cellular functions by phosphorylating and activating the AGC-kinase Gad8 (AKT or SGK1 in human cells) at its hydrophobic motif (HM) and its turn motif (TM). Full activation of Gad8 requires additional phosphorylation at the activation loop (AL) by Ksg1 (PDK1 in human cells) (22, 23, 24, 25).

Crosstalk between TOR signaling and DNA damage response pathways has been noted by multiple studies (26), however, the mechanism underlying the sensitivity of TORC2-Gad8 mutant cells to DNA damage or DNA replication stress is poorly understood. Cells defective in the DNA damage checkpoint (*e.g.*, cells disrupted for *rad3*^+^) fail to inhibit entrance into mitosis, and quickly die when exposed to genotoxic stresses (27). Our previous studies demonstrated that TORC2-Gad8 delays cell cycle progression in response to genotoxic stresses (14), indicating that the mechanism underlying genotoxic stress sensitivity in TORC2-Gad8 mutant cells is distinct from that of cells lacking the DNA damage checkpoint kinases. It is likely that the sensitivity of TORC2-Gad8 to DNA damaging conditions involves defects in DNA damage repair, as TORC2-Gad8 mutant cells accumulate DNA damage (Rad52) foci and show synthetic lethality with mutations in the homologous recombination (HR) pathway (17).

Studies have shown that TORC2-Gad8 has a great impact on transcriptional regulation, under both normal and stress conditions. In response to replication stress, TORC2 is required for transcriptional upregulation of the MBF (*Mlu*I cell-cycle box-binding factor)-regulated genes (28), which likely contributes to DNA replication stress sensitivity (29). On the other hand, under normal growth conditions, TORC2-Gad8 contributes to gene silencing at subtelomeric regions and the mating-type locus (29). Thus, TORC2-Gad8 is involved both in the positive and negative regulation of transcription. Intriguingly, Gad8 physically interacts with proteins associated with active chromatin, including subunits of the SAGA complex, Gcn5, Ubp8, Spt7 (30) and Taf12 (20), subunits of the Paf1C complex, Paf1, Leo1, and the BET bromodomain protein Bdf2 (30). Disruption of SAGA subunits, including the acetyltransferase Gcn5, or the de-ubiquitinase Ubp8, or disruption of the BET bromodomain protein Bdf2, restore transcriptional activation of MBF-regulated genes and gene silencing (30). Thus, it appears that a fine balance is required between specific positive regulators of transcription (SAGA and Bdf2) and TORC2-Gad8 to regulate transcription processes (30).

One of the earliest and most conserved events in the DNA damage response is the phosphorylation of the SQ motif on the C-terminal tail of histone H2A, known as γH2A in yeast cells and γH2AX in higher eukaryotes (31). H2AX and H2A phosphorylation is carried out by the ATM and ATR DNA damage checkpoint kinases, and their counterparts in yeast cells, respectively (32, 33, 34). Direct phosphorylation is initiated at the double-strand DNA break (DSB) or at stalled or collapsed DNA replication fork sites and then spreads to nearby chromatin (35, 36, 37). γH2A/γH2AX serves as a platform for the recruitment of DNA repair proteins, although additional, overlapping mechanisms to recruit DNA repair proteins, also exist (33, 34, 38, 39). γH2A/γH2AX also contributes to chromatin mobility and organization (40). γH2AX may be removed from the chromatin by the action of protein phosphatases (41) or by histone exchange (42).

Here we demonstrate that TORC2-Gad8 is required for inducing γH2A in *S. pombe* in response to exposure to DNA damage in growing cells, as well as in quiescence in the absence of external DNA damaging agent. In addition, we show that the loss of transcription and chromatin regulators restores γH2A induction in TORC2 mutant cells. Our genetic analysis suggests that TORC2-Gad8 does not regulate γH2A by regulating Rad3 or Tel1 activity; rather, it may affect chromatin structure and function in a way that inhibits γH2A accumulation.

## Results

### TORC2-Gad8 is required for induction of γH2A in response to DNA damage

**Figure 1:**
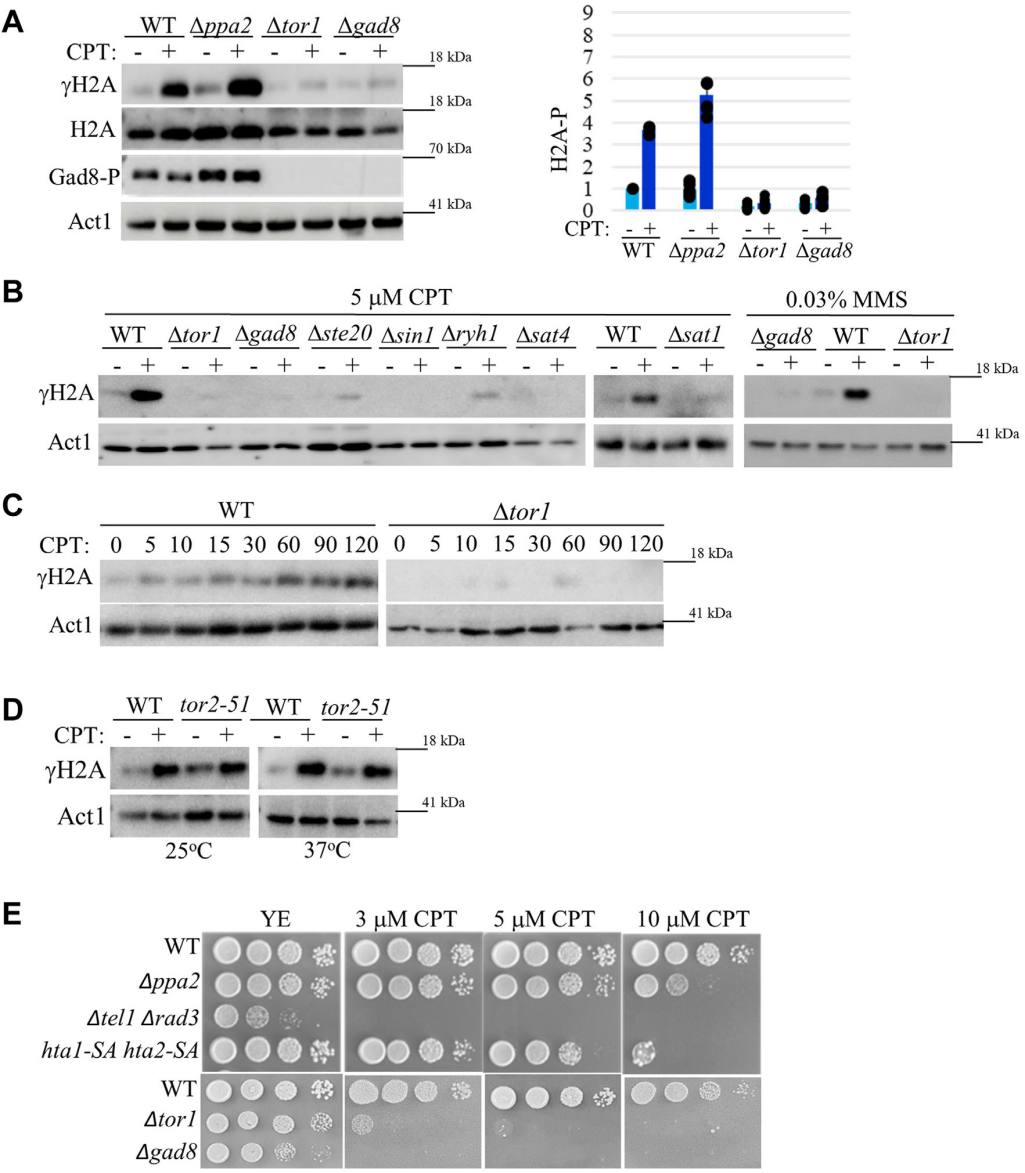
**TORC2-Gad8 is required for induction of γH2A in response to DNA damage.***A*, Tor1 and Gad8 are required for induction of γH2A in response to camptothecin (CPT). The indicated strains were grown in rich medium (YE) to mid-log phase, re-suspended in fresh medium, and exposed for 1 h to 5 μM CPT (+) or left untreated (−). Immunoblots were analyzed with phospho-specific anti-γH2A antibody, anti-H2A and anti-actin as loading controls, and anti-Gad8-S546P antibody (Gad8-P) as a readout for TORC2 (Tor1) activity. The *right panel* shows the quantification of three independent biological repeats. *B*, the TORC2 complex and its upstream regulators are required for the induction of γH2A. Cells were grown as above, re-suspended in fresh medium, and treated for 1 h with 5 μM CPT or 0.03% MMS, as indicated. *C*, kinetic experiments following induction of γH2A in response to 5 μM CPT for 120 min in wild-type and Δ*tor1* cells. Samples were taken for analysis at the indicated time points (minutes). *D*, TORC1 is not required for induction of γH2A. Cells carrying the temperature-sensitive mutation *tor2-51* were grown to mid-log phase, re-suspended in fresh medium, and shifted to 25 °C (permissive temperature) or 37° (restrictive temperature) for 4 h, followed by treatment with 5 μM CPT for 1 h. *E*, mutations in TORC2-Gad8, but not in the DNA damage checkpoint phosphorylation sites in *hta1*^*+*^ and *hta2*^*+*^, result in severe DNA damage sensitivity. Serial dilution of exponentially growing wild-type and deletion mutant cells were spotted onto rich medium, with no drug (YE), or in the presence of 3,5, or 10 μM CPT.

*S. pombe* contains two histone H2A genes that encode C terminal SQE motifs, *hta1*^*+*^ and *hta2*^*+*^. To gain further insight into the functional role of TORC2-Gad8 in DNA damage responses, we tested the induction of H2A phosphorylation (γH2A) in deletion mutant cells lacking components of TORC2-Gad8 signaling. To induce DNA damage, we used camptothecin (CPT), which induces collisions between advancing replication forks and topoisomerase I, leading to S-phase-specific double-strand breaks (43). Remarkably, we found very little, or no induction of γH2A in response to 5 μM of CPT in cells disrupted for the catalytic subunit of TORC2, *tor1*^+^, or its downstream substrate, the AGC kinase encoded by *gad8*^+^ (Fig. 1*A*). The total level of H2A is only slightly reduced in Δ*tor1* or Δ*gad8* cells compared with wild-type cells (Fig. 1*A*), indicating that it is not a reduction in the total level of H2A that accounts for the low level of γH2A in Δ*tor1* or γH2A Δ*gad8* mutant cells. Disruption of the type 2A phosphatase *ppa2*^+^ led to a slight increase in γH2A (Fig. 1*A*). This finding suggests that in *S. pombe* PP2A does not play a prominent role in regulating γH2A, unlike the role of PP2A in regulating γH2A in mammalian cells (41). Disruption of the unique subunits of TORC2, Δ*ste20* or Δ*sin1*, or disruption of the upstream regulators of TORC2, Δ*ryh1*, Δ*sat4* or Δ*sat1* (25), resulted in no γH2A induction in response to CPT (Fig. 1*B*), consistent with a role for TORC2-Gad8 in inducing γH2A. In addition, Δ*tor1* or Δ*gad8* cells did not show induction of γH2A in response to methyl methane sulfonate (MMS), another DNA-damaging drug, which acts through DNA alkylation (Fig. 1*B*, right panel). Kinetic experiments revealed that in wild-type cells γH2A becomes detectable at around 5 min following exposure to 5 μM CPT and its intensity increases for at least 2 h (Fig. 1*C*). In contrast, in Δ*tor1* cells no induction of γH2A is observed even 2 h following exposure to CPT (Fig. 1*C*). Please note that while we were able to obtain reliable results with one batch of antibodies against total H2A (Fig. 1*A*), using other batches, or several different brands of anti-H2A antibodies, we failed to obtain reliable and reproducible results. Hence, hereafter, we used anti-actin antibody as a loading control.

To explore whether TORC1 is required for inducing γH2A, we used a temperature-sensitive allele of *tor2*^+^, the gene encoding the catalytic subunit of TORC1 (10). At the restrictive temperature, *tor2-51* cells induce γH2A similar to the induction observed in wild-type cells (Fig. 1*D*). Thus, TORC2, but not TORC1, is required for induction of γH2A in response to DNA damage.

It was previously reported that *S. pombe* cells in which the phosphorylation sites at the C terminal SQE of *hta1* and *hta2* were replaced with residues that cannot be phosphorylated, *hta1-S129A hta2-S128A*, show only mild DNA damage sensitivity (34). The mild sensitivity of *hta1-S129A hta2-S128A* to DNA damage is likely due to alternative mechanisms for the recruitment of DNA repair proteins, the main role attributed to γH2A (33, 34, 38, 39). Consistent with previous results, we found that *hta1-S129A hta2-S128A* strains are far less sensitive to DNA damage compared with Δ*tel1* Δ*rad3* strains (Fig. 1*E*). Cells disrupted for *ppa2*^+^ also displayed mild sensitivity to DNA damage (Fig. 1*E*), possibly due to the role of PP2A-Pab1 in regulating cell cycle progression (26). As previously reported (14, 17, 18, 28, 30), TORC2-Gad8 mutant cells were highly sensitive to DNA damage stresses. Notably, Δ*tor1* or Δ*gad8* cells show sensitivity to CPT at a concentration as low as 3 μM, while *hta1-S129A hta2-S128A* cells show sensitivity only at 10 μM CPT (Fig. 1*E*). Thus, although the lack of γH2A in TORC2-Gad8 mutant cells is dramatic, it cannot account for the severe DNA damage stress sensitivity of TORC2-Gad8 mutant cells.

### TORC2-Gad8 is required for induction of γH2A in quiescence

**Figure 2:**
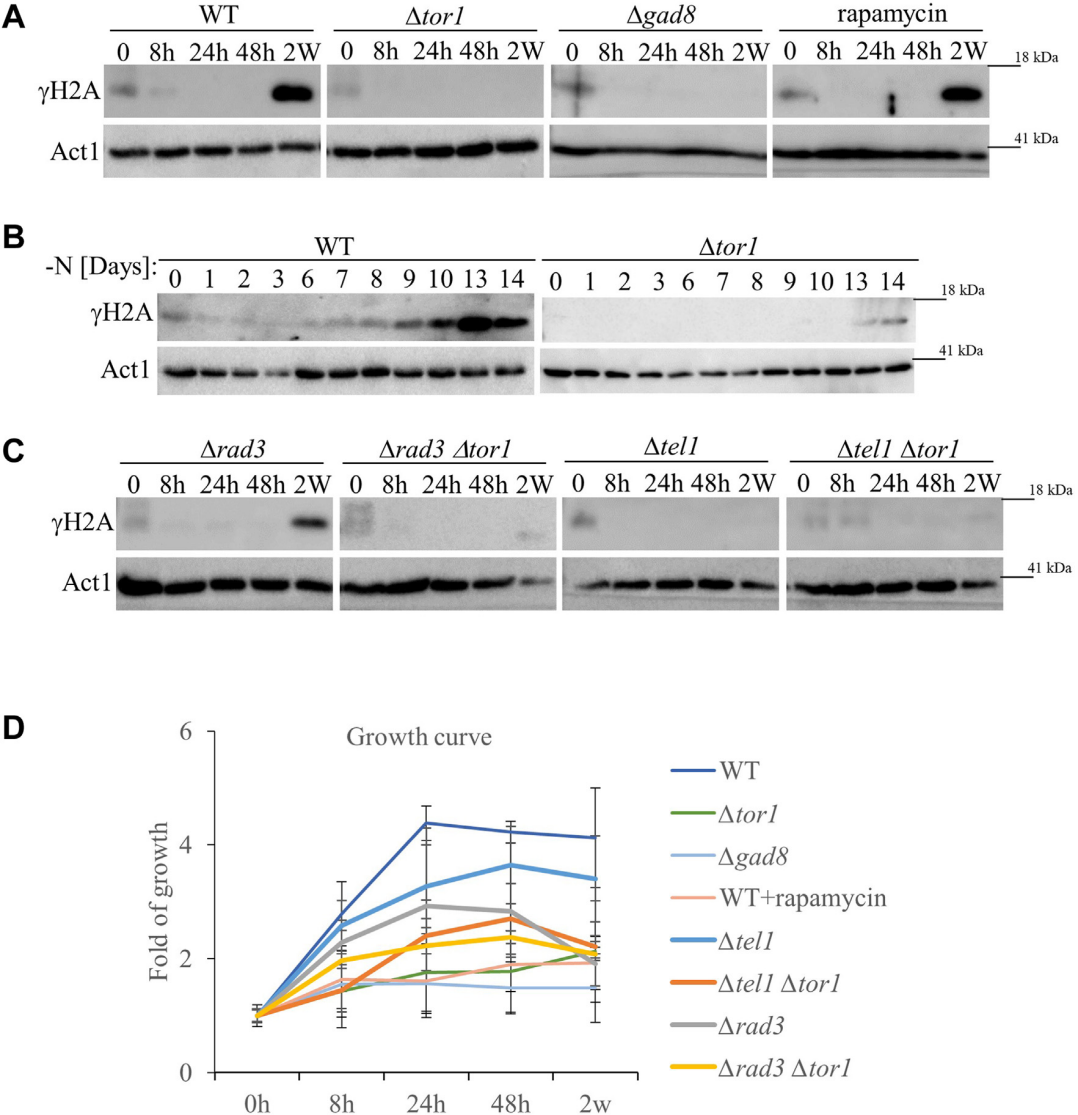
**TORC2-Gad8 is required for the induction of γH2A in quiescence.***A*, Tor1 and Gad8 are required for γH2A accumulation in quiescence. Immunoblot analysis. Cells were grown in minimal medium (EMMGlu) to mid-log phase before being washed and re-suspended in nitrogen-free medium (EMM-N), Samples were taken at mid-log phase (0) and after re-suspension in EMM-N for 8, 24 and 48 h (8 h, 24 h, 48 h), and 2 weeks (2 w). *B*, kinetics of accumulation of γH2A in quiescence. Cells were grown to mid-log phase and starved for nitrogen as described above. Samples were taken for analysis at mid-log phase (0), and then at the indicated following days. Immunoblots were analyzed with anti-γH2A antibody and anti-actin as loading control. *C*, Tel1 but not Rad3 is required for γH2A in quiescence. The indicated strains were grown, starved, sampled and immunoblots were analyzed as described in *A*. *D*, growth curves of fold change of colony forming units (CFU). The indicated strains were grown to mid-log phase in minimal (EMMGlu) medium and were then re-suspended in nitrogen-starvation medium (EMM-N). Cells were sampled at the indicated times and cell viability was determined by determining CFU on a complete (YE) medium. Average fold changes in CFU of at least three independent experiments at each time point were plotted.

TORC2 phosphorylates its downstream Gad8 kinase at serine 546, within the highly conserved hydrophobic motif (HM) (23, 24). Previously, we found that Gad8-S546 phosphorylation (Gad8-S546P) is lost 24- or 48-h following nitrogen-starvation (early quiescence), but re-appears in 2 weeks following nitrogen-starvation (late quiescence) (44). While monitoring TORC2-Gad8 activities during quiescence, we found that wild-type cells induce γH2A in late quiescence (2 weeks in nitrogen starvation), without exposure to an external DNA damaging agent (Fig. 2*A*, leftmost panel). This is the first time, to our knowledge, that induction of γH2A is recorded in quiescent *S. pombe* cells. As in growing cells exposed to DNA damage, Tor1 or Gad8 are required for the induction of γH2A in quiescence (Fig. 2*A*, two middle panels). Rapamycin, which inhibits several *S. pombe* TORC1-dependent functions (45, 46, 47, 48), does not affect γH2A in quiescence (Fig. 2*A*, rightmost panel), consistent with our finding that TORC1 is not required for γH2A (Fig. 1*D*). Kinetic experiments, following the induction of γH2A for 2 weeks in quiescent cells, indicate that γH2A starts to accumulate on day 9 and is further increased on days 10 to 14, while no induction of γH2A is observed in Δ*tor1* cells (Fig. 2*B*). We next explored the roles of the DNA damage checkpoint kinases in inducing γH2A in quiescence. Interestingly, we found that the loss of Rad3 does not affect the level of γH2A in quiescence, while the loss of Tel1 alone abolishes γH2A (Fig. 2*C*). Double mutant cells, Δ*rad3* Δ*tor1* or Δ*tel1* Δ*tor1* showed no γH2A accumulation, as expected (Fig. 2*C*). Viability count demonstrated that the mutant strains examined mostly maintained their viability throughout the experiment (Fig. 2*D*). Note that upon nitrogen starvation, wild-type cells divide approximately twice, before arresting growth, while cells lacking Tor1 or Gad8 fail to execute the two rounds of divisions in the absence of a nitrogen source, as previously noted (5).

In conclusion, TORC2 is required for the accumulation of γH2A in quiescence. Unlike the overlap activities of Tel1 and Rad3 in inducing γH2A during the logarithmic phase in response to DNA-damaging drugs (34), Tel1, but not Rad3, is required for γH2A in quiescent cells.

### Tel1 is involved in phosphorylating Gad8 at the hydrophobic motif (HM) in quiescent cells

**Figure 3:**
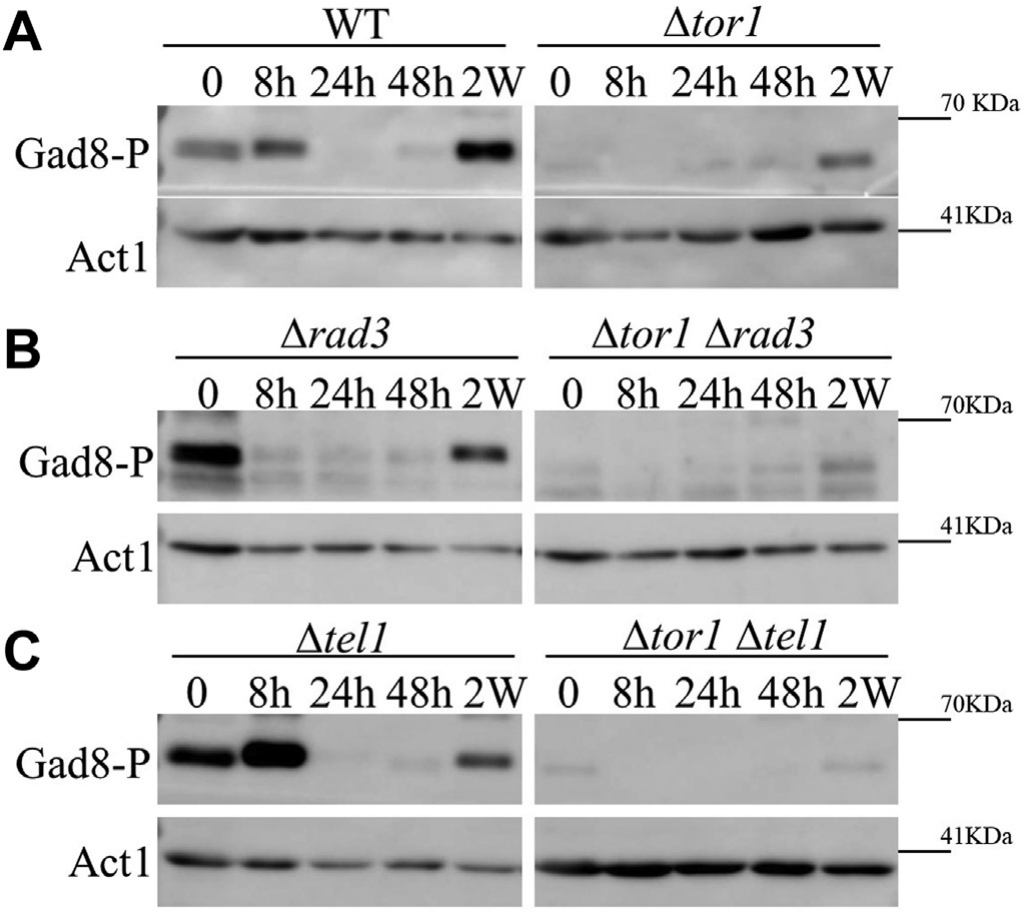
**Tel1 is involved in phosphorylation of Gad8 at its hydrophobic (HM) motif.***A–C*, cells were grown in minimal medium (EMMGlu) to mid-log phase before being washed and re-suspended in nitrogen-free medium (EMM-N), Samples were taken at mid-log phase (0) and after re-suspension in EMM-N for 8, 24 and 48 h (8 h, 24 h, 48 h), and 2 weeks (2 w). Immunoblot analysis with custom-made antibody against the phosphorylated form of Gad8 at its HM motif, S546 (Gad8-P). Anti-actin antibody was used for loading control.

The hydrophobic motif in Gad8, Gad8-S546, is a well-conserved TORC2-dependent phosphorylation site. As previously shown (23, 24), Gad8-S546P is lost during early quiescence but re-appears in late quiescence (Fig. 3*A*, left panel). Surprisingly, we found that in Δ*tor1*, Gad8-S546 remains phosphorylated in late quiescence (Fig. 3*A*, right panel). This finding suggests that there is another kinase, in addition to Tor1, capable of phosphorylating the HM of Gad8. We thus explored the possibility that Rad3 or Tel1, which belong to the same family of proteins as TOR, may contribute to Gad8-S546P in quiescence. We found that the absence of Rad3 does not affect Gad8-S546P (Fig. 3*B*). In contrast, the loss of Tel1 abolishes the phosphorylation of Gad8-S546 in late quiescence in Δ*tor1* cells (Fig. 3*C*). This finding suggests that Tor1 and Tel1 have an overlapping function in inducing phosphorylation of Gad8-S546 in quiescence. One possibility is that Tel1 directly phosphorylates Gad8-S546. Indeed, the mammalian DNA-PK kinase, another member of the PIKK protein kinases, which has no orthologue in *S. pombe*, directly phosphorylates AKT (orthologue of Gad8) at the hydrophobic motif in response to insulin (49). Alternatively, Tel1 may induce another kinase to phosphorylate Gad8-S546. Using *in vitro* kinase assays, we have failed so far to detect phosphorylation of Gad8 by Tel1. Thus, whether Tel1 directly or indirectly affects the phosphorylation of Gad8-S546 remains unknown. In any case, our findings suggest that Tel1 plays a dual role in the induction of γH2A in quiescence: directly phosphorylating H2A (Fig. 2*C*) and inducing Gad8 activity through Gad8-S546P (Fig. 3*C*), which in turn, is required for accumulation of γH2A.

### Torin1 abolishes induction of γH2A in response to DNA damage

The use of selective inhibitors of TOR is a means to examine whether the effect of TORC2 on γH2A is immediate. We used Torin1, a selective ATP-competitive inhibitor of TOR kinases, which inhibits both TORC1 and TORC2 in higher eukaryotes and in *S. pombe* cells (50). We found that treatment of wild-type *S. pombe* cells with Torin1 for 30 min prior to the addition of 5 μM CPT abolishes the induction of γH2A in response to CPT (Fig. 3*A*, lane 4). This finding suggests that the effect of loss of TOR activity on the accumulation of γH2A is not the result of an adaptive mechanism, but that it is rather a direct effect. We detected no induction of γH2A in *tor2-G2040D* mutant cells, which carry a mutation in Tor2 that renders it resistant to Torin1 (50). This finding is consistent with our analyses demonstrating that TORC2 alone is responsible for the regulation of γH2A (Fig. 1, *A* and *D*).

**Figure 4:**
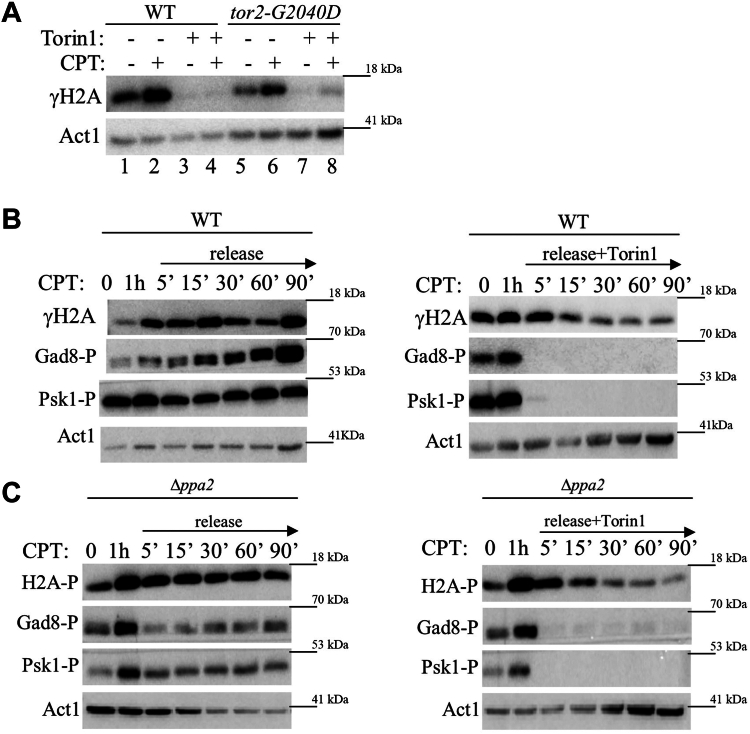
**Torin1 abolishes the induction of γH2A in response to DNA damage.***A*, Torin1 abolishes γH2A in wild-type and in *tor2-G2040D* (Torin1-resistant *tor2*) mutant cells. Cells were grown to mid-log phase in rich (YE) medium, re-suspended in fresh medium, and treated with 25 μM Torin1 for 30 min (+), followed by the addition of 5 μM CPT for 1 h (+), or left untreated (−). Immunoblots were analyzed with anti-γH2A antibodies. Anti-actin antibody was used for loading control. *B* and *C*, Torin1 accelerates the loss of γH2A following release from camptothecin (CPT). Wild type (*B*) or Δ*ppa2* (*C*) cells were grown to mid-log phase, sampled (0), re-suspended in fresh medium, and then treated with 5 μM CPT for 1 h and re-suspended in fresh YE medium without, or with 25 μM Torin1. Cells were sampled and proteins were extracted at the indicated time points (in minutes). Immunoblots were analyzed for γH2A, phosphorylation of the HM of Gad8 (Gad8-P), as a readout for TORC2 activity, and phosphorylation of the HM motif of Psk1 (Psk1-P), as a readout for TORC1 activity. Anti-actin antibody was used for loading control.

We next used Torin1 to determine the kinetics by which loss of TORC2 affects the rate of loss of γH2A following release from DNA damaging conditions. Cells were induced to phosphorylate H2A with 5 μM CPT for 1 h before being released into a fresh medium, either in the absence or presence of 25 μM Torin1. Without treatment of Torin1, no loss of γH2A signal is observed for 1.5 h after release from CPT (Fig. 4*B*, left panel). In contrast, upon release into Torin1-containing medium, a reduction in γH2A is observed already 15 min following release, a further reduction is observed following incubation of 30 min, and thereafter the level of γH2A remains constant (Fig. 4*B*, right panel). Thus, Torin1 reduces the γH2A signal after a relatively short time but does not eliminate it. In comparison, the phosphorylation of Psk1 and Gad8, which are direct targets for phosphorylation by TORC1 and TORC2, respectively, are completely lost already 5 min following the release into Torin1 containing medium (Fig. 4*B*, right panel). Disruption of Ppa2 does not affect the kinetics of loss of γH2A, either in the absence or presence of Torin1 (Fig. 4*C*), supporting our suggestion that Ppa2 in *S. pombe* does not play a prominent role in regulating the levels of γH2A (see above, Fig. 1*A*).

Taken together, our findings indicate that inhibition of TORC2 by Torin1 decreases γH2A relatively quickly, but not as quickly as the loss of phosphorylation of direct targets of TOR complexes. Thus, our findings are in accord with a scenario in which TORC2 does not directly phosphorylate H2A, but its activity is required to maintain high levels of γH2A.

### Loss-of-function mutations in transcription and chromatin modifier factors restore γH2A in TORC2 mutant cells

**Figure 5:**
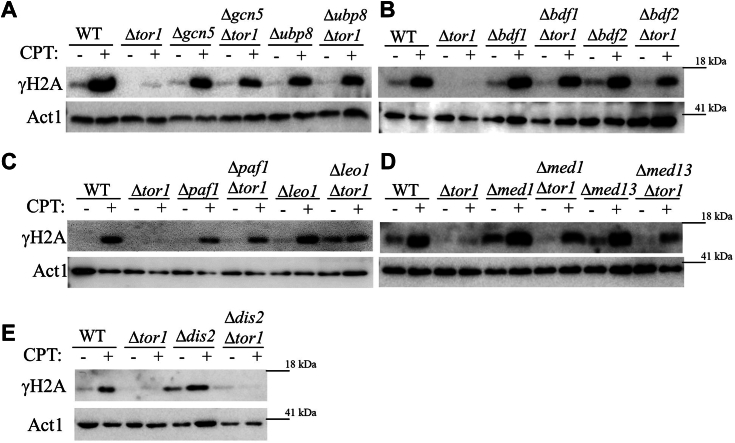
**Deletion mutations in transcription and chromatin modification genes restore γH2A in cells lacking Tor1 (TORC2).***A–E*, the indicated strains were grown to mid-log phase in rich medium (YE) before being exposed for 1 h to 5 μM CPT (+), or left untreated (−). Immunoblots were analyzed with phospho-specific anti-γH2A antibodies. Anti-actin antibody was used for loading control.

We previously identified several physical interactors of Gad8, including members of the SAGA complex (Gcn5 and Ubp8), the BET bromodomain proteins (Bdf2), and members of the Paf1C complex (Paf1 and Leo1) and the Mediator complex (Med1) (30). We also found that disruption of all of the above Gad8 interactors, as well as another BET bromodomain proteins, Bdf1, restored gene silencing at subtelomeric genes in a manner dependent on the accumulation of H3K9 methylation. Disruption of SAGA components, or disruption of *bdf2*^+^, also suppressed the sensitivity of TORC2 mutant cells to DNA replication (HU) or DNA damage (CPT) stresses (30). Here we found that disruption of SAGA components (Δ*gcn5*, Δ*ubp8*) disruption of Δ*bdf1* or Δ*bdf2*, disruption of Paf1C components (Δ*paf1*, Δ*leo1*) or disruption of Mediator components (Δ*med1*, Δ*med13*) restore γH2A induction in Δ*tor1* cells (Fig. 5, *A*–*D*). The SAGA complex, Bdf1/Bdf2, Paf1C, and Mediator are involved in positive regulation of transcription and chromatin modifications, suggesting that the lack of γH2A in Δ*tor1* cells is linked to a role in TORC2 transcription and/or chromatin structure. Dis2, the catalytic subunit of PP1 (protein phosphatase 1), was shown to regulate transcription, in particular the transition from transcription elongation to termination (51). Since our analysis points at a possible link between transcription regulation, chromatin state, and accumulation of γH2A, we examined the possible effects of disruption of *dis2*^+^ on the induction of γH2A. Δ*dis2* cells showed similar induction of γH2A to that observed in wild-type cells, and no suppression of the lack of γH2A in Δ*tor1* cells (Fig. 5*E*). Thus, our data do not support at present the involvement of PP1 phosphatases in the regulation of γH2A.

## Discussion

One of the earliest responses to double-strand breaks is the induction of histone H2A/H2AX phosphorylation (γH2A) by DNA damage checkpoint kinases (32, 33, 34), known as Rad3 (ATR) and Tel1 (ATM) in *S. pombe* (34). Here, we demonstrated that TORC2, containing the catalytic subunit Tor1, and the immediate effector, the AGC kinase Gad8, are required for induction of γH2A in response to DNA damaging agents (CPT or MMS). Unexpectedly, we found that *S. pombe* cells also induce γH2A in late quiescence (2-weeks nitrogen-starved cells), in the absence of external genotoxic stress. Induction of γH2A in quiescence requires Tel1 and Tor1 (TORC2), but not Rad3. Disruption of the SAGA complex (Δ*gcn5* or Δ*ubp8*), Paf1C complex (Δ*paf1* or Δ*leo1*), Mediator complex (Δ*med1*) or the Bdf1/2 genes (Δ*bdf1* or Δ*bdf2*), previously identified as mutations that suppress loss of gene silencing at subtelomeric regions (30), also suppress the lack of induction of γH2A in TORC2 mutant cells.

Several factors are thought to affect the level of γH2A induction in response to DNA damage: the level of activity of the DNA damage checkpoint kinases, ATM and ATR, in human cells, the level of de-phosphorylation of γH2A, which is thought to be controlled by PP2A complexes in human cells (41) and chromatin remodeling (42). In human cells, PP2A is also thought to affect γH2A by regulating the activity of ATM (52). In *S. pombe*, loss of both Rad3 and Tel1 leads to complete loss of γH2A, while loss of only Rad3 or Tel1 leads to a moderate reduction in γH2A (34). No phosphatase responsible for de-phosphorylation of γH2A has yet been identified in *S. pombe*. Two *S. pombe* genes encode for the catalytic subunit of PP2A complexes, *ppa1*^+^ and *ppa2*^+^. Of these Ppa2 is the major PP2A phosphatase, and is expressed at much higher levels compared with Ppa1 (53). Our findings showing similar levels of induction, or loss, of γH2A in wild-type and Δ*ppa2* mutant cells (Figs. 1*A* and 4*C*), do not support a direct role of Ppa2 in de-phosphorylation γH2A or a major effect on the activity of Rad3 or Tel1.

The possibility that TORC2-Gad8 affects γH2A by regulating Rad3/Tel1 activities is also unlikely. Previously, we showed that Chk1, a direct substrate of Rad3, is only very slightly reduced in TORC2-Gad8 mutant cells (14, 17), suggesting that the activity of Rad3 is not reduced in TORC2 mutant cells. In addition, Δ*rad3* cells advance into mitosis and fail to arrest cell cycle progression in response to DNA damage, while TORC2-Gad8 mutant cells properly delay mitosis in response to DNA damage. The possibility that the loss of γH2A in TORC2-Gad8 mutant cells is due to the downregulation of Tel1 is also unlikely, since the loss of TORC2-Gad8 results in a more dramatic effect of loss γH2A, compared with the effect of loss of Tel1. In addition, our finding indicates that Tel1 and TORC2 act in parallel, at least with respect to the phosphorylation of Gad8, the substrate of TORC2 (Fig. 2*C*).

Interestingly, chromatin remodelers affect the levels of γH2A. Thus, for example, the Ino80 and Swr1 chromatin remodeling enzymes antagonistically regulate γH2A *via* promoting the incorporation of γH2A and the histone variant Htz1, respectively (42). The possibility that TORC2-Gad8 affects chromatin remodeling is intriguing in view of the roles already attributed to TORC2-Gad8 in chromatin modification, in particular, in the spreading of the heterochromatic marker H3K9me at subtelomeric regions and the mating-type locus (29). Both the defect in γH2A induction or heterochromatin spreading in TORC2-Gad8 mutant cells are suppressed by the disruption of Paf1C, SAGA, or Bdf1/2 (30). Loss of Paf1C in *S. pombe* cells restricts histone turnover, leading to increased spreading of H3K9me (54). Paf1C cooperates with Epe1, a putative histone demethylase, to restrict heterochromatin spreading (30), while Epe1 specifically recruits both SAGA and Bdf2 (55, 56). Thus, it is tempting to speculate that the mechanism by which Paf1C, SAGA and Bdf1/2 restore γH2A and H3K9me involves Paf1C-SAGA-Bdf1/2-dependent chromatin remodeling. Further studies are required to better characterize the role of TORC2-Gad8 in chromatin structure and function, including possible roles in nucleosome biogenesis, nucleosome assembly, and specific modification of the chromatin around DSBs.

γH2AX/γH2A is typically associated with induced DSBs but is also formed in the absence of external DNA damage, in a manner that may not involve the formation of DSBs [reviewed in (57, 58)]. Thus, for example, γH2AX/γH2A is formed in response to stalled or collapsed replication forks (59, 60), or in mitotic cells in the absence of external DNA damage (61, 62). During normal replication in *S. pombe* cells, γH2A is found at replication fork barriers and breakage sites, as well as at heterochromatic regions, where γH2A is associated with the establishment heterochromatin by the histone H3K9 methyl transferase Clr4 (36). In mammalian cells, senescence is a physiological condition in which phosphorylation of H2AX occurs in the absence of external DNA damage (63, 64). Our findings that quiescent *S. pombe* cells induce γH2A in a manner dependent on TORC2-Gad8 and Tel1, but not on Rad3, is intriguing, and calls for further investigation. It has been shown that DNA lesions accumulate in quiescent cells (65), but whether these or other chromosomal changes are responsible for the induction of γH2A in late quiescence in *S. pombe* cells, is yet to be explored.

Finally, γH2AX is used as a biomarker to estimate the level of DNA damage and to analyze DNA damage signaling (66). Our finding that TORC2-Gad8 signaling is required for γH2A formation may be conserved in evolution and thus relevant for studying DNA damage responses in cancerous cells. Indeed, in human breast cancer cells, treatment with the pan-mTOR inhibitor PP242 reduced γH2AX foci formation, most likely *via* inhibiting mTORC2 and not mTORC1 (67). Other studies show that inhibition of mTORC1 causes increased levels γH2AX (68, 69), although mTORC1-S6K1 was also shown to stimulate high levels of γH2AX in response to DNA damage (70). Given the effects of mTOR on γH2AX and the major roles of mTOR in promoting cancer, understanding the interplay between mTOR and DNA damage responses is important for the interpretation of γH2AX levels in cancerous cells and for developing strategies for cancer therapy.

## Experimental procedures

### Yeast strains, media, growth, and starvation assays

*S. pombe* strains used in this paper are listed in the Table S1. Experiments were performed using early exponential cells unless otherwise specified. Yeast cells were cultured in rich YE medium supplemented with adenine and uracil, or in Edinburgh Minimal Medium, EMM (5 g/liter NH4Cl, 2% glucose), or EMMGlu (EMM containing 3.75 g/L glutamic acid instead of ammonia as a nitrogen source), as specified. When minimal media was used, it was supplemented with amino acids according to the auxotrophic requirements, as described in (71). Cell cultures were incubated at 30 °C, unless otherwise specified. When using temperature-sensitive alleles, cells were grown at 25 °C before re-suspending the cells in a fresh medium and shifting them to the restrictive temperature of 37 °C. For nitrogen-starvation experiments, prototrophic cells were grown in EMMGlu (EMM containing 3.75 g/L glutamic acid instead of ammonia as a nitrogen source) to mid-log phase, sampled, and then washed three times with distilled water before being re-suspended in EMM medium lacking any nitrogen source (EMM-N). Samples were removed at the indicated time for protein extractions and live cell count. No glucose was added during the nitrogen-starvation period. Rapamycin was added at the final concentration of 100 ng/ml, camptothecin (CPT) at 5 μM (unless otherwise specified), methylmethanesulfonate at 0.03% and Torin1 at 25 μM. Before adding the indicated drug, cells were centrifuged and re-suspended in fresh media. Control cultures were treated the same, without adding the drug. For spot assays, cells were grown to early logarithmic phase in rich (YE) medium, serially diluted, and spotted on the indicated plates. Plates were incubated at 30 °C.

### Genetic manipulation and strain construction

Gene deletions were performed by standard PCR-based methods (72). Oligonucleotides used for gene deletions are listed in Table S2. Double mutant strains were constructed either by genetic crosses or by the introduction of a disruption cassette into the required genetic background.

### Protein extraction and Western blotting

Total protein extracts for analysis by polyacrylamide gel electrophoresis were prepared from 2 to 4 × 10^8^ cells. Cells were grown to logarithmic phase, centrifuged and the pellets were resuspended in 200 μl of cold 20% trichloroacetic acid. After the addition of the same volume of glass beads, cells were disrupted by vortexing for 15 min. Glass beads were washed twice with 200 μl of cold 5% trichloroacetic acid, and the resulting extract was spun for 10 min at 13,000 rpm in an Eppendorf microcentrifuge at 4 °C. The pellet was resuspended in 200 ml of sample buffer X2, neutralized by adding 100 μl of 1 M Tris base, heated to 80 °C for 5 min, and finally centrifuged at 13,000 rpm for 20 s 5 μl were analyzed by polyacrylamide gel electrophoresis in the presence of sodium dodecyl sulfate (SDS-PAGE).

Proteins were resolved by SDS-PAGE 10 to 15% acrylamide gels and transferred to nitrocellulose membranes, blocked with 5% milk in TBST (0.05% Tween). For analyses of γH2A, membranes were immunoblotted with anti-histone H2A antibody (phospho S129) (ab17353, Abcam), anti-histone H2A antibody (39235, active motif), and anti-actin (691001, MP). The phosphorylated form of Gad8 at S546 (HM) was detected by custom-made antibodies, described in (73). Psk1 phosphorylation was detected using anti-phospho-p70S6 kinase Thr389 (Cell Signaling, catalog #9206). Detection was carried out using the ECL SuperSignal detection system (Thermo Scientific). Western blots were quantitatively analyzed using ImageJ software.

## Data availability

All data is contained with the manuscript.

## Supporting information

This article contains supporting information.

## Conflicts of interest

The authors declare that they have no conflicts of interest with the contents of this article.
