# Supplementary material for: TORC2 is required for the accumulation of γH2A in response to DNA damage
Source: J Biol Chem. 2024 Jul 4;300(8):107531. doi: 10.1016/j.jbc.2024.107531 (PMC11321321; doi:10.1016/j.jbc.2024.107531)
Supplement: Supporting information [file mmc1.docx]

**TORC2 is required for accumulation of γH2A in response to DNA damage**

Adiel Cohen, Lea Lubenski, Ava Mouzon, Martin Kupiec and Ronit Weisman

**Supporting Information:**

Table S1

Table S2

**Table S1. Strains used in this study**

| **Strain** | **Genotype** | **Source** |
| --- | --- | --- |
| TA0000 | 972 *h*^-^ | P Fantes lab |
| TA0001 | 975 *h*^+^ | P Fantes lab |
| TA0002 | *leu1-32 ura4-D18 ade6-M210 h^-^* | P Fantes lab |
| TA0003 | *leu1-32 ura4-D18 ade6-M210 h*^+^ | P Fantes lab |
| TA0824 | *tel1::LEU2 leu1-32 ade6 h*^+^ | JP Cooper lab |
| TA1252 | *ryh1::kanMX6 leu1-32 h^-^* | K Shiozaki lab |
| TA1253 | *sat1::kanMX6 leu1-32 h^-^* | K Shiozaki lab |
| TA1254 | *sat4::kanMX6 leu1-32 h^-^* | K Shiozaki lab |
| TA1274 | *tor2-51:ura4^+^ ura4-D18 ade6-M210 h^+^* | S Moreno lab |
| TA2893 | *gcn5::kanMX6 leu1-32 ura4-D18 ade6-M216 h^90^* | Lab stock |
| TA3033 | *paf1::kanMX6 leu1-32 his2 ura4-DS/E ade6 h^+^* | Lab stock |
| TA3073 | *tor1::ura4^+^ paf1::kanMX6 leu1-32 ura4 ade6* | Lab stock |
| TA3511 | *gad8:: kanMX6 h^+^* | Lab stock |
| TA3520 | *tel1::kanMX6 tor1::ura4^+^ h^+^* | Lab stock |
| TA3527 | *tor1::kanMX6 h^+^* | Lab stock |
| TA3570 | *rad3::kanMX6 tel1::hphMX6 h^+^* | Lab stock |
| TA3852 | *leo1:: hphMX6* | Lab stock |
| TA3875 | *tor1:: hphMX6 leo1:: kanMX6* | Lab stock |
| TA3891 | *bdf2::hphMX6 leu1-32 ura4-D18 ade6-M210 h^-^* | Lab stock |
| TA3907 | *bdf2::hphMX6 tor1::ura4^+^leu1-32 ura4 ade6* | Lab stock |
| TA3908 | *bdf1::hphMX6 tor1::ura4^+^ leu1-32 ura4 ade6* | Lab stock |
| TA3959 | *ubp8::hphMX6 leu1-32 ura4-D18* | Lab stock |
| TA3498 | *tel1:: kanMX6 h^+^* | Lab stock |
| TA4025 | *gcn5::kanMX6 tor1::ura4^+^ leu1-32 ura4-D18 ade6-M216 h^90^* | Lab stock |
| TA4176 | *med13::hphMX6 leu1-32 ura4-D18 ade6-M210 h^-^* | This study |
| TA4190 | *med1::hphMX6 leu1-32 ura4-D18 ade6-M210 h^-^* | Lab stock |
| TA4205 | *med13::KanMX6  leu1-32 his2 ura4 ade6 tor1::ura4^+^* | This study |
| TA4219 | *med1::hphMX6  tor1::ura4^+^ leu1-32 ura4-D18 ade6* | This study |
| TA4544 | *tor1::ura4^+^ ubp8::hphMX6* | Lab stock |
| TA4845 | *hta1-S129A::ura4^+^ hta2-S128A::his3^+^ leu1-32 ura4-D18 ade6-210 his3-D1 h^+^* | N. Boddy lab |
| TA5140 | *tor2-G2040D h^+^* | J Petersen lab |
| TA5299 | *sin1::kanMX6 leu1-32 ura4-D18 ade6-M210 h^+^* | Lab stock |
| TA5300 | *ste20::kanMX6 leu1-32 ura4-D18 ade6-M210 h^+^* | Lab stock |

**S2 Table. Oligonucleotides used for gene deletions**

| **Gene deleted** | **Name of primer** | **Sequence** |
| --- | --- | --- |
| *gad8::kanMX6* | #682 F | TTAAAAGAAAAGATAGAGGGAAAGCGAGCTTTTAAAAATCAGTTCATTTTTTTTTTCTACTCCAAACAGACGTTACCGAACGGATCCCCGGGTTAATTAA |
|  | #683 R | ATGTAAAAGAGGCAAGAAAAGCGGCATGTATGAGTAAAAATGAGAAAACTTTCAAAATAAACAAAGAAGTGTCAAATTCAACATTTGATTAAAATAGAAC |
| *tor1::kanMX6* | #1367 F | ATTGTGATGAATGCCTAAGTGGAAGAATTGAACACCGCGACTATTAGAAAGTCTATCGTTTCACTCGCTCTCTTTGATTCCGGATCCCCGGGTTAATTAA |
|  | #1368 R | CAGAAACGAGCGAATTTATAGACATAAATTAATAACAACACGAAAAAAATTATCATAATCTCAAAAAACAGAAAACATCAGAATTCGAGCTCGTTTAAAC |
| *tel1::hphMX6* | #1455 F | CCTTTTACTCAATTATCAGTTTACGCCAAATATGTTAATATATTAATGAAGAAAAGATAACTTTTACTCGATGTAATACTCGGATCCCCGGGTTAATTAA |
|  | #1456 R | TGCATTTCGTTTCACTAATTGCCAAGAGGTACGAATTAATTAGAAAGAAAACACTCATGAAAATAAATTAACTATATCGCGAATTCGAGCTCGTTTAAAC |
| *rad3::hphMX6/ kanMX6* | #1458 F | ATAAATGCTCAAGACTTTGAACGCGCGTGTTGCGTTTTAAAAAGGCCTTTTTTTGAATTGAATCAATGGTTTGATATAGTCGGATCCCCGGGTTAATTAA |
|  | #1459 R | TAATAAATAAAATATCTTCGATTCAAATCATAAGTTTAATAATGGGTAGCTTGTTCATTGAAATTTTTGTTAGTAAAATGGAATTCGAGCTCGTTTAAAC |
